# Selective Efficacy of Static and Dynamic Imagery in Different States of Physical Fatigue

**DOI:** 10.1371/journal.pone.0149654

**Published:** 2016-03-01

**Authors:** Thiago Ferreira Dias Kanthack, Aymeric Guillot, Leandro Ricardo Altimari, Susana Nunez Nagy, Christian Collet, Franck Di Rienzo

**Affiliations:** 1 Université de Lyon, Université Claude Bernard Lyon 1, Laboratoire Interuniversitaire de Biologie de la Motricité (LIBM), F-69622, Villeurbanne, France; 2 Grupo de Pesquisa em Sistema Neuromuscular e Exercício, Londrina State University, Paraná, Brazil; 3 CAPES Foundation, Ministry of Education of Brazil, Brasília, Distrito Federal, Brazil; 4 Institut Universitaire de France, Paris, France; 5 Unidad de Fisioterapia, Universidad de Alcalá, Alcalá de Henares, Madrid, España; University of Minnesota, UNITED STATES

## Abstract

There is compelling evidence that motor imagery contributes to improved motor performance, and recent work showed that dynamic motor imagery (dMI) might provide additional benefits by comparison with traditional MI practice. However, the efficacy of motor imagery in different states of physical fatigue remains largely unknown, especially as imagery accuracy may be hampered by the physical fatigue states elicited by training. We investigated the effect of static motor imagery (sMI) and dMI on free-throw accuracy in 10 high-level basketball athletes, both in a non-fatigued state (Experiment 1) and immediately after an incremental running test completed until exhaustion (20m shuttle run-test–Experiment 2). We collected perceived exhaustion and heart rate to quantify the subjective experience of fatigue and energy expenditure. We found that dMI brought better shooting performance than sMI, except when athletes were physically exhausted. These findings shed light on the conditions eliciting optimal use of sMI and dMI. In particular, considering that the current physical state affects body representation, performing dMI under fatigue may result in mismatches between actual and predicted body states.

## Introduction

Motor imagery is the mental representation of an action without physical execution of the corresponding movement. Experimental data provides ample evidence that motor imagery contributes to enhanced motor performance in both sporting and everyday life motor skills [1–3]. Likewise, motor imagery has been shown to promote motor recovery in injured athletes and in patients suffering from motor disorders [4–7]. Motor imagery further positively affects psychological factors involved in high-level sport performance, e.g. motivation and focus [8, 9]. Due to structural and functional similarities with physical practice (PP), motor imagery can operate on neurophysiological processes mediating motor learning, i.e., activity-dependent neuroplasticity [7, 10]. Additionally, MI can be used to control non-invasive brain-computer interfaces ([11], for a recent overview). Typically, brain signals recorded during various MI tasks are classified and transduced into electric commands to control a robotic device (e.g., [12, 13]). These technologies emphasizes that brain activity during MI embeds relevant components of the corresponding motor tasks [14, 15].

Nonetheless, imagery guidelines and instructions for effective interventions vary to a great extent across disciplines (sports, music, education, medicine and psychology) (for a review, see [3]). While most training frameworks recommend to directly combine motor imagery with PP during actual training sessions [16, 17], little is known about the optimal guidelines of motor imagery practice, particularly considering that PP may elicit physical fatigue. The effect of fatigue upon motor imagery might change athletes’ dispositions towards mental rehearsal. So far, physical fatigue is known to impair motor performance, but recent work suggested that it might also impair imagery accuracy [18, 19], although this deleterious effect is not systematically observed [20]. Recently, Rozand, Lebon [21] reported that mental fatigue has sufficient potential to alter the temporal organization of motor imagery, without necessarily impairing muscle performance (see [22, 23]). Interestingly, there is yet no experimental data comparing the respective effects of different MI content upon different states of physical fatigue.

Several theoretical frameworks were proposed to determine the optimal way to perform imagery and develop effective interventions [16, 17, 24, 25]. Practically, motor imagery should match the spatial and temporal parameters of the corresponding action to achieve optimal transfer from mental representation to actual performance [26, 27]. However, whether adequately embedding motor imagery into actual training sessions—where physical fatigue can be prevalent—has been far less considered. According to its classical definition, MI should be performed without concomitant body movements. Yet, recent modes of MI practice combine MI with actual body movements (e.g., [28]). Such body movements are of limited amplitude, i.e., insufficient to fully mimic the imagined action, but nonetheless sufficient to embody its temporal invariants (e.g., skiers reproducing with hands movements the timing of their slalom according to the curves of the ski slope, while concomitantly imagining their own performance). Guillot, Moschberger [29] investigated the efficacy of dynamic motor imagery (dMI) practice matching the patterns of the high jump. The dMI intervention improved both imagery quality and motor performance. This experiment directly addressed, through dMI, the inter-relationships between mental and motor processes to boost the outcome of imagery interventions. Other experimental studies had showed that dMI might contribute to improve motor performance, vividness and confidence of athletes [28], as well as the temporal congruence between actual and imagined actions [30]. While the effects of fatigue on static MI (sMI) mentioned earlier have already been explored, little is known about its effect on dMI and subsequent consequences on motor performance. A related issue of interest is therefore to investigate whether high loads of physical exercise eliciting a physical fatigue state might interfere with both sMI and dMI quality and thus motor performance. Practically, physical fatigue could limit the efficacy of sMI and dMI due to interference between actual and predicted body states [18, 19].

Free-throw is a basketball skill of specific importance due to its complexity and its crucial role on final result, particularly when the score is tight. This role is exacerbated during the last minutes of the match, when players are exhausted [31]. sMI has extensively been shown to improve performance in basketball free-throw shooting [32–34]. The immediate effects of both sMI and dMI on free-throw performance (e.g., during matches) have not yet been investigated. Past protocols foremost implemented sMI practice periods from 1 day (with free-throw performance the next day [32]) to 3 weeks [35], but the effect of practicing sMI and dMI under fatigued states has never been considered. As previously mentioned, physical fatigue is known to alter physical performance, but might interfere with the capacity of imagining oneself performing an action as well [18, 19]. Fatigue presumably elicits erroneous updates of the internal representation of the action due to the central integration of proprioceptive feedback under altered body state [19, 36, 37]. In the first experiment, we investigated whether sMI and dMI might elicit short-term effects on free-throw shooting accuracy in non-fatigued athletes. Then, we tested the efficacy of sMI and dMI in the same sample of athletes, under a state of physical fatigue corresponding to the last minutes of basketball games.

## Experiment 1

### Material and Methods

#### Participants

Ten state-level male basketball players (*M* = 18.4 years, *SD* = 0.5; *M* = 7.3 years of practice, *SD* = 2.3; 6 hours/day of training, 5 days per week) voluntarily participated in the study. They provided an informed written consent in agreement with the terms of the Declaration of Helsinki (1982). The study was approved by the ethical committee of Londrina State University (Brazil).

#### Experimental design

The study took place in an indoor court meeting the international standards for line distance, hoop height and ball weight. Each experimental session occurred at the same time of the day for each condition (9 am ± 1 hour).

Before taking part in the experiment, athletes completed a 4 week sMI program for familiarization, including 2 sessions of 5 min per week embedded in the classical course of regular training. We delivered a limited amount of sMI practice to meet the purpose of familiarization without interfering with the classical course of training. We basically wanted athletes to have basic knowledge of sMI before engaging in the main experiment, hence preventing novelty bias. We intended to familiarize athletes with the different modalities of sMI practice of basketball skills. Athletes were requested to mentally rehearse strategic schemata of their teammates, shooting and passing actions using the first or third person perspective. They only used the first person visual perspective during the first week, combined first person visual perspective and kinesthetic information during the second and third weeks, and only kinesthetic MI during the last week of familiarization. Athletes were systematically instructed to match the spatial and temporal characteristics of the sequences.

#### Procedure

**Fig 1:**
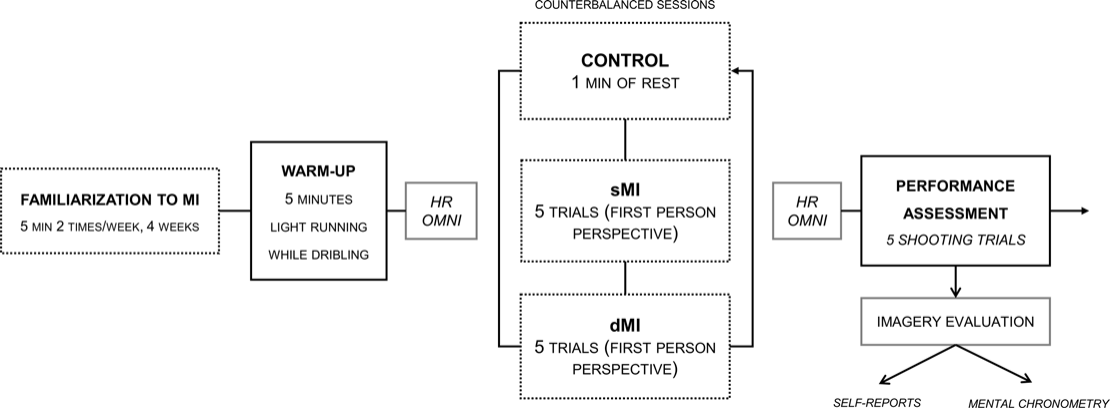
Flowchart of the experimental design. sMI = Static Motor Imagery, dMI = Dynamic Motor Imagery, HR = Heart Rate, OMNI = Self-exhaustion perception, TL = Training Load from previous day. sMI, dMI and Shooting time measured in each condition.

After familiarization to sMI, athletes went through three experimental conditions (i.e., one time each). Experimental sessions were scheduled within a span of 10 days: *i)* sMI condition where athletes performed five MI trials of the shooting task using the first-person perspective, before performing five actual free-throws; *ii)* dMI condition where athletes completed five imagery trials while performing concurrently slight body movements matching the pattern of the shooting task (i.e., limited body movements which do not result in the completion of the task during its imagination, but sufficient to allow an embodiment of the temporal invariants of the task such as the durations of the preparatory/shooting phases of free throw shooting), using the first-person perspective. dMI was followed by five actual free-throws; *iii)* control condition without any imagery intervention (CONTROL), where athletes remained motionless, discussing their daily training with one of the experimenters for an amount of time corresponding to that allocated to sMI and dMI. This control condition was followed by five actual free-throws. The purpose of having five free-throws for each condition was twofold. First, we wanted to limit the learning/habituation effect. The repeated performance of this closed skill in athletes might have produced ceiling effects which would interfere with the possible gains originating from a preliminary practice of sMI/dMI [33]. Second, we wanted athletes to feel a context which met as much as possible the demands of the free-throws performed during actual basketball games. Usually, players have two consecutive free-throws, sometimes three according to the type of defensive foul, and must immediately perform at a high level of accuracy. In the present experimental design, we increased this number to 5 to increase sample size. The experimental conditions were provided in a counterbalanced order across participants to control carryover effects. To quantify the perceived carryover training load from one experimental session to another, athletes were presented before each session the modified Borg Scale [38] (Fig 1). Finally, each condition was separated from the 2 others by exactly 72 hours. Each started by a warm-up of five minutes involving running and dribbling with a ball between cones at own self pace.

#### Imagery interventions

During sMI and dMI, athletes were instructed to imagine as accurately as possible, the free-throw sequence from receiving the ball up to the final phase of shooting. During dMI, they were required to perform slight arm movements and semi-flexion of the legs thus miming the temporal invariants of the actual task. Athletes were positioned in the context of actual free-throw, i.e. on the line in front of the hoop. This was expected to facilitate mental simulation [39]. As indices of both sMI and dMI quality, participants self-reported the level of perceived vividness on a Likert scale ranging from 1 (“*Unclear and inaccurate mental representation*”) to 6 (“*Perfectly clear and vivid mental representation*”). They also held a manual timer in the non-dominant hand measuring the time needed to imagine a free-throw sequence, from receiving the ball up to the final shooting phase. We then calculated the delta between actual and imagined free-throw durations as an index of temporal congruence [40].

#### Shooting accuracy

All trials were recorded with a video camera, to evaluate the number of successful trials and the duration of each free-throw through the number of images between the two action boundaries. Performance was evaluated in terms of converted free-throws (from 0 to 5).

#### Heart Rate and Exertion Perception

During each experimental session, athletes held a cardiac monitor (Polar FT2^®^) measuring the heart rate (HR) in beats per minute (bpm). HR was collected immediately after warm-up and sMI/dMI/CONTROL trials, under each condition. We used the OMNI Scale to measure fatigue perception after the warm-up, and after intervention of each experimental session [41].

#### Statistical Analyses

We used R [42] and *lme4* [43, 44] to build a mixed linear model for each dependent variable (*i*.*e*., shooting accuracy, heart rate and OMNI ratings). We entered the experimental conditions as fixed effect, (*i*.*e*., dMI, sMI and CONTROL). As a random effect, we had intercepts by participants (i.e., intra-subject analysis). We applied a rank transformation to the dependent variables of interest, in order to increase statistical power [45]. For corrected post-hoc comparisons [46], we iterated the mixed linear model on datasets from which classes of the factor considered were removed to allow dual comparisons. For HR and OMNI data, the recording moment was added as factor of the model (pre- and post-intervention) (Fig 1). The alpha threshold was settled at 5%. Considering the stringent statistical model implemented for data analysis, trends were investigated using the Smallest Worthwhile Change (SWC) [47]. SWC was developed to assess performance changes in sports, and specifically the percentage of chance that a given measure is considered Superior/Trivial/Inferior under two conditions. SWC provides a probability for each class according the following ranges: < 1% for “almost impossible”; 1–5% for “very unlikely”, 5–25% for “unlikely”, 25–75% for “possible”, 75–95% for “probably”, 95–99% for “very likely” and >99% for “certain”.

### Results

#### Physical fatigue

Data from one participant was not included due to technical failure. All athletes reported similar levels (*M* ± SD) of perceived fatigue before each experimental session on the Borg Scale (4.2 ± 2.5, χ^2^(2) = 0.039, *p* = 0.98). OMNI ratings after warm-up and intervention were comparable across conditions without time effect (pre = 2.2 ± 1.9, post = 1.1 ± 1.8, *p* = 0.79). Mixed linear models yielded a CONDITION * TEST interaction for HR (χ^2^(2) = 0.019, *p* = 0.01). HR values were similar across conditions before intervention (131 ± 15 bpm, *p* = 0.22). However, HR values after dMI (114 ± 14 bpm) were higher compared to both sMI and CONTROL (96 ± 12 bpm and 93 ± 10 bpm, respectively; *p* = 0.02).

#### sMI/dMI accuracy

**Fig 2:**
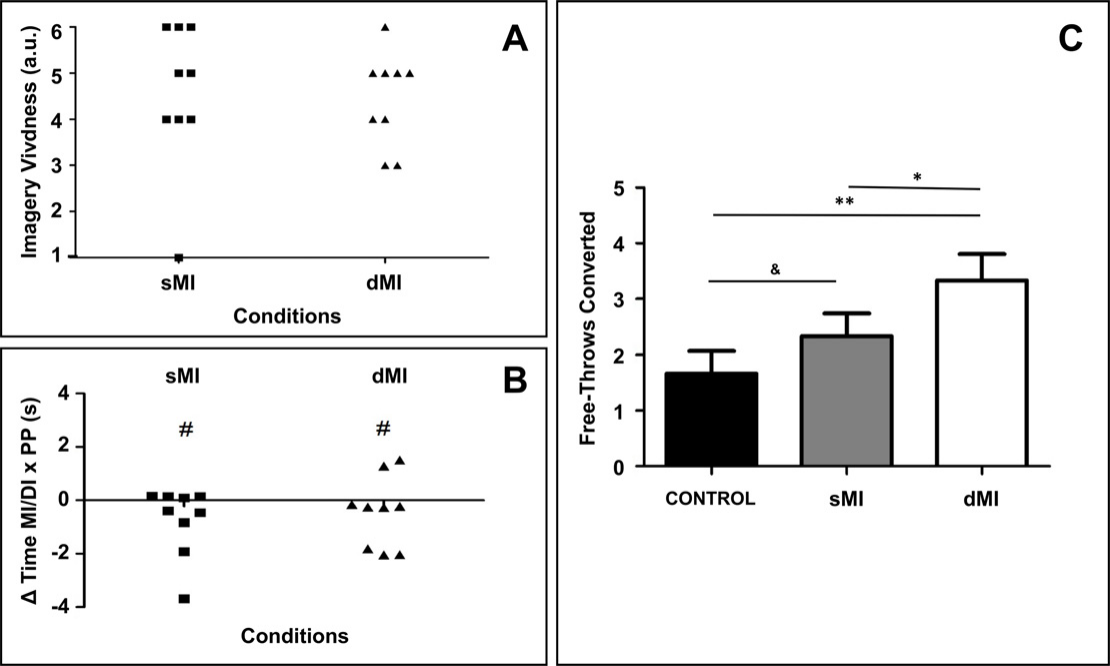
A. Self-reports of imagery vividness. B. Delta time between actual and imagined durations for sMI and dMI. C. Free-throw shooting accuracy for each athlete under each experimental condition. * p<0.05; ** p<0.01; ^#^ Different from 0, p<0.05, ^&^ Statistical trend (0.05<p<0.1).

No difference was found when comparing sMI and dMI vividness (4.5 ± 1.2) and the delta between actual and imagined free-throw durations (Fig 2). At the group level, athletes underestimated actual durations during both sMI and dMI by 0.61 ± 1.2 s (*p* < 0.01).

#### Shooting accuracy

Mixed linear modeling yielded a main effect of experimental conditions on shooting accuracy (χ^2^(2) = 12.01, *p* = 0.002). Shooting accuracy was higher following dMI (3.3 ± 1.4 free-throws converted) compared to both sMI (2.3 ± 1.2, p<0.05) and CONTROL (1.6 ± 1.2, p<0.001) (Fig 2). A trend towards higher shooting accuracy during sMI compared to CONTROL was also observed (*p* = 0.06). This was confirmed by the SWC analyses, which supported a probable beneficial effect of sMI over CONTROL (i.e., 79% Superior, 17% Trivial, and 4% Inferior).

## Experiment 2

### Material and Methods

#### Participants

The same participants as in experiment 1 took part in experiment 2 (18.4 ± 0.5 years; 7.3 ± 2.3 years of practice, 6 hours/day of training 5 days per week). They provided a new informed written consent in agreement with the terms of the Declaration of Helsinki (1982). The study was approved by the ethical committee of Londrina State University (Brazil).

#### Experimental design

Experiment 2 took also place in the same indoor court as during experiment 1. The experimental intervention occurred at the same time of day for each condition (9 am ± 1 hour) to avoid circadian effects. We implemented a counterbalanced design in order to control carryover effects between the experiments and conditions of the paradigm.

#### Experimental conditions

The design involved three experimental sessions, which athletes went through once. Experimental sessions were scheduled within a span of 10 days and separated from each other by exactly 72 h. There was therefore a total intervention time of 20 days for experiments 1–2. Immediately after the warm-up (same content as in Experiment 1), the participants completed an incremental running test until exhaustion [48]. They were then subjected to one of the 3 following conditions: *i)* sMI under fatigue (sMI_f_) where imagery trials (*n* = 5) preceding the 5 shooting trials were completed immediately after exhaustion; *ii)* dMI under fatigue (dMI_f_), allowing slight movements related to the real task during motor imagery where imagery trials (*n* = 5) preceding the 5 free throws were also performed immediately after exhaustion; *iii)* control under fatigue (CONTROL_f_) where athletes remained motionless and talked with the experimenter about their daily training during an equivalent amount of time, after exhaustion. Experimental sessions were delivered in a counterbalanced order to prevent carryover effects. Participants rated their perceived exertion before each experimental session on the modified Borg Scale [38] for each experimental session.

#### Exhaustion Test

Athletes performed a shuttle test requesting running between two cones separated from each other by 20 m [48]. Athletes ran from one cone to the other according to the auditory pace of a metronome. The metronome first paced a running speed of 8 km/h, and increased every minute by 0.5 km/h. The test ended in case of complete exhaustion, or if when a participant failed to match the metronome pace 3 times in a row, the test was over and he was considered under fatigue. This test is known as closely reproducing efforts encountered during basketball games.

#### sMI_f_/dMI_f_ intervention

For sMI_f_ and dMI_f_, athletes were positioned on the free-throw line for better mental simulation [39]. They were instructed to combine first-person visual imagery with kinesthetic imagery during sMI_f_. For dMI_f_, slight arm movements as well as semi-flexions were allowed. Immediately after the intervention, we collected the perceived vividness of sMI_f_/dMI_f_ on a Likert scale ranging from 1 (“*Unclear and inaccurate mental representation*”) to 6 (“*Perfectly clear and vivid mental representation*”). We also collected sMI_f_/dMI_f_ durations, and calculated the delta between actual and imagined durations, a reliable index of temporal congruence [40].

#### Heart Rate and Exertion perception

**Fig 3:**
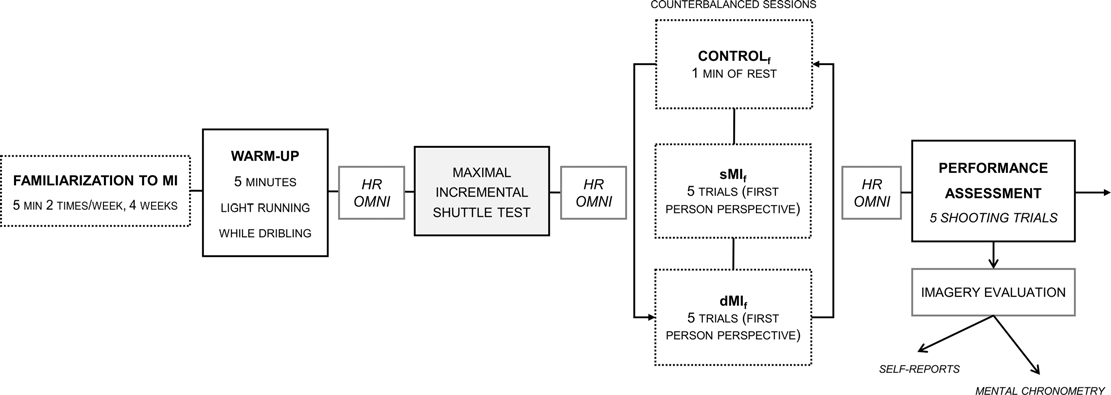
Experimental paradigm for Experiment 2 and dependent variables. CON = Control, sMI_f_ = Static Motor Imagery under fatigue, dMI_f_ = Dynamic Motor Imagery under fatigue, HR = Heart Rate, OMNI = Self-exhaustion perception, TL = Training Load from previous day. sMI_f_, dMI_f_ and Shooting times were measured in each condition.

Athletes held a cardiac monitor (Polar FT2^®^). HR (bpm) was collected after warm-up, after the exhaustion test, and immediately after the experimental intervention. Participants rated their perceived level of fatigue on the OMNI Scale after warm-up, after the exhaustion test and after the experimental intervention (Fig 3).

#### Free-throw performance

Actual shooting times (from the moment participants received the ball up to the final phase of shooting), as well as the number of converted free throws (from 0 to 5), were collected to evaluate shooting accuracy.

#### Statistical analyses

We implemented the same statistical procedure as in Experiment 1, based on mixed linear models. For HR and OMNI data, we included the recording moment (post warm-up, pre- and post-intervention) as a factor of the model, in addition to the experimental condition factor (sMI_f_, dMI_f_ and CONTROL_f_). The alpha threshold was settled at 5%. We also applied the SWC approach [47] as in Experiment 1.

### Results

#### Physical fatigue

**Fig 4:**
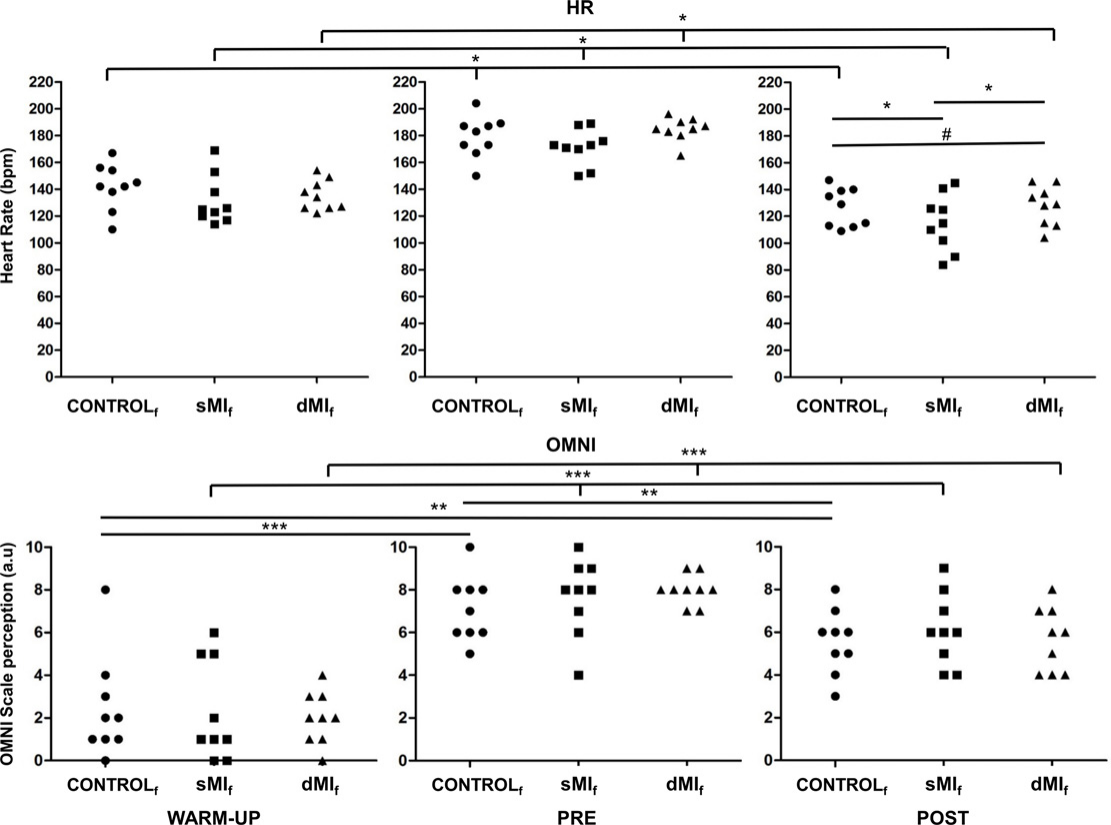
HR values and OMNI score after warm-up, pre-intervention and post-experimental intervention. * p<0.05, ** p<0.01, *** p<0.001, ^#^ Statistical trend (0.05 < p < 0.1).

All players reported similar levels of perceived fatigue before each experimental session on the Borg Scale (χ^2^(2) = 0.061, *p* = 0.96; sMI_f_: 4.3 ± 2.5, dMI_f_: 4.2 ± 1.5, CONTROL_f_: 4.4 ± 1.5). We observed no difference among conditions related to HR and OMNI after the warm-up (HR: χ^2^(2) = 3.91, *p* = 0.14, OMNI: χ^2^ (2) = 0.91, *p* = 0.93) and post-exhaustion test (HR: χ^2^(2) = 5.29, *p* = 0.07, OMNI: χ^2^(2) = 4.43, *p* = 0.10). As well, OMNI revealed no difference between post-intervention conditions (χ^2^(2) = 1.14, *p* = 0.56) (Fig 4). Lower HR values were recorded (χ^2^(2) = 7.54, *p* = 0.02) after the sMI_f_ session (131 ± 18 bpm) as compared to those monitored under CONTROL_f_ and dMI_f_ sessions (141 ± 17 bpm and 135 ± 15 bpm, respectively, *p* < 0.05). Values of HR in dMI_f_ tended to be lower compared to CONTROL_f_ (*p* = 0.09) (Fig 4).

Both HR and OMNI values significantly increased between the warm-up and the end of the exhaustion test preceding the experimental intervention (HR: χ^2^(1) = 83.25, *p* < 0.001, OMNI: (χ^2^(1) = 67.70, *p* < 0.001; Warm-up HR: 123 ± 17 bpm, OMNI: 2.2 ± 1.9; Pre-intervention HR: 178 ± 13 bpm, Pre-intervention OMNI: 7.5 ± 1.4). A significant decrease after the experimental intervention was then recorded (HR: χ^2^(1) = 65.86, *p* < 0.001, OMNI: χ^2^ (1) = 27.37, *p* < 0.001; Post-intervention HR: 136 ± 15 bpm, Post-intervention OMNI: 5.7 ± 1.5) (Fig 4).

#### Shooting accuracy

**Fig 5:**
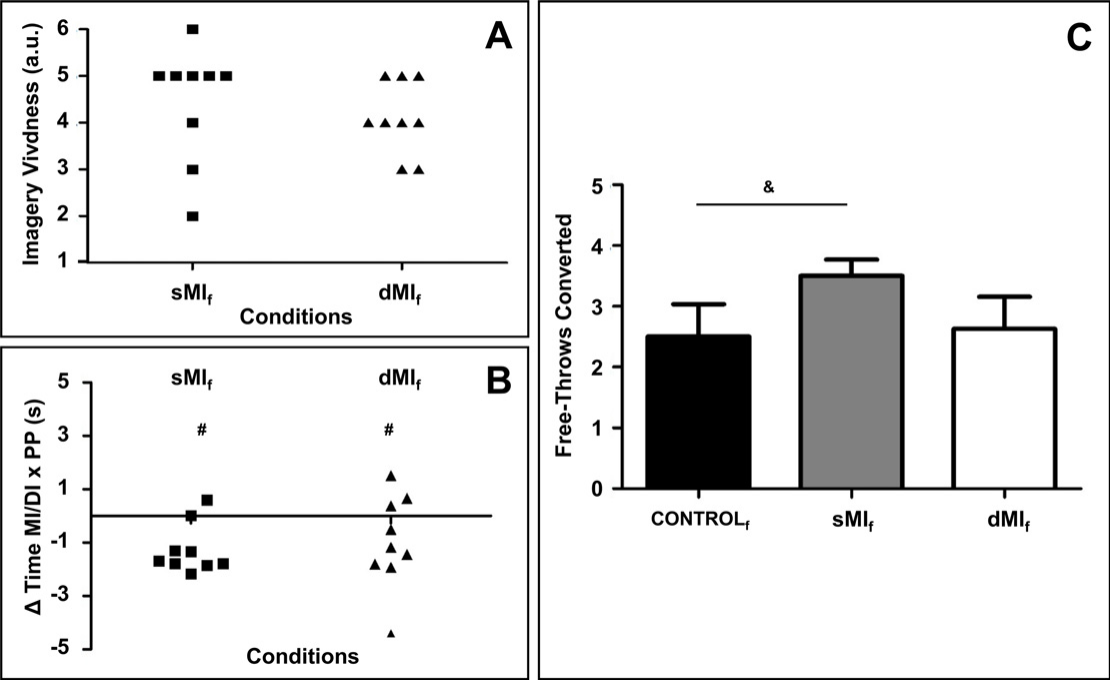
A. Self-reports of imagery vividness. B. Delta between actual and imagined free-throw durations during sMI_f_ and dMI_f_ conditions. C. Free-throw accuracy across conditions. ^#^ Different from 0 (p<0.05), ^&^ Statistical trend (0.05 < p < 0.1).

Self-reports of imagery vividness (sMI_f_: 4.4 ± 1.2, dMI_f_: 4.1 ± 0.7) and the delta between actual and imagined durations were similar across conditions (χ^2^(1) = 0.62, *p* = 0.43, and χ^2^(1) = 0.29, *p* = 0.58 respectively, Fig 5). As in Experiment 1, participants overall underestimated actual durations by 1.1 ± 1.3 s (*p* < 0.01).

Mixed linear modeling yielded a trend for experimental conditions on the free-throw accuracy (sMI_f_: 3.5 ± 0.7, dMI_f_: 2.6 ± 1.5, CONTROL_f_: 2.5 ± 1.5 free-throws converted, χ^2^(2) = 2.78, p = 0.09, Fig 5). Shooting accuracy tended to be higher during sMI_f_ as compared to CONTROL_f_ (*p* = 0.08). The SWC analysis revealed that sMI_f_ presented a higher percentage of chance to yield greater shooting accuracy values as compared to those recorded during both dMI_f_ (88% Superior, 10% Trivial, and 2% Inferior) and CONTROL_f_ (83% Superior, 14% Trivial, and 3% Inferior).

## Discussion

The present study was designed to evaluate the selective efficacy of sMI and dMI under different states of fatigue. Overall, dMI was found to have the most beneficial effect on free-throw performance (Experiment 1), except when athletes were physically exhausted (Experiment 2), a physical state making sMI more efficient.

First, OMNI data showed that participants were in a similar state of perceived exhaustion before engaging in the different conditions for both Experiments 1 and 2. On average, the participants perceived a “moderate” level of fatigue. HR data further revealed that they adequately complied with the experimental instructions, particularly during Experiment 2 where a neat HR increase attested high energy expenditure following the shuttle test. HR data also showed that before completing the experimental conditions, participants were in a similar state of physiological arousal. During Experiment 1, we recorded higher HR values immediately after dMI as compared to both sMI and CONTROL. Indeed, dMI included slight body movements simultaneously with mental representation. This brings an added amount of physical activity potentially contributing to higher levels of cardiac activity. During Experiment 2, we recorded lower HR values under sMI_f_ as compared to both CONTROL_f_ and dMI_f_. Motor imagery practice is known to temporarily reduce cardiac activity due to the involvement of attentional resources (see [49]). However, the intake-rejection theory early postulated different HR changes according to the focus of attention [50]. Briefly, the authors reported decreased HR when the participants were requested to process external information, whereas they observed increased HR when the participants focused their attention on internal cues. Our results may seem different from what the theory postulated. Yet, although MI is considered an internal process, the content of mental representation is externally and spatially oriented. This suggests that the HR decrease under sMI_f_ may reflect the focus of attentional resources on the environmental context of the free-throw. This effect might have been emphasized compared to experiment 1 as players exhibited increased levels of cardiac activity immediately after the maximal incremental test. Such MI-related effects occurred to a lesser extent under dMI_f_ since they were possibly counterbalanced by overt body movement concomitant to motor imagery. From a physiological level of analysis, changes in HR attest to concurrent effects of the parasympathetic and orthosympathetic branches of the autonomic nervous system. We can thus consider that both systems were co-activated when high concentration level was associated with high energy expenditure. Although we did not record physiological data in the present study, recent findings related to autonomic nervous system functioning have demonstrated that the parasympathetic branch could be activated through the nucleus ambiguus which was demonstrated to specifically reduce heart rate activity in case of high cognitive demand (see [51, 52]). This remains a working hypothesis, awaiting further experimental investigation.

An important issue addressed in the present study is whether practicing sMI or dMI was likely to elicit short-term effects on shooting accuracy. In a study involving high-level junior race standard skiers, Callow, Roberts [28] reported higher levels of vividness during dMI than during sMI, hence supporting the potential benefits of dMI in applied sport settings. The positive effects of dMI on motor performance were recently confirmed by Guillot, Moschberger [29] in a sample of high jumpers, while Fusco, Iosa [30] later reported higher levels of temporal congruence between PP and dMI rather than between PP and sMI while imagining locomotors sequences.

Data from Experiments 1 and 2 provided evidence that no deleterious effects occurred under sMI or dMI. Interestingly, higher shooting accuracy was recorded in the dMI condition for Experiment 1 and the sMI_f_ condition for Experiment 2. Although we support that sMI contributed to increase basketball performance [33], findings from Experiment 1 confirmed better efficacy from dMI [28, 29], and further demonstrated that dMI can improve immediate subsequent motor performance when players are not physically fatigued. Jackson, Lafleur [53] underlined three key components involved in motor performance improvement over time: *i)* declarative knowledge, which refers to the explicit information about the skill available to the participant, *ii)* infra-conscious processes referring to implicit knowledge (procedural memory) related to the skill that participants are able to implement during motor processing but cannot verbally describe (e.g., complex muscle synergies, etc.), and *iii)* the feedback loop of actual skill execution which participants use to correct and stabilize motor programs. Based on this theoretical framework, sMI involves the two first aspects (i.e., declarative knowledge and infra-conscious processes), whereas dMI might involve the three aspects of the model, hence providing additional benefits.

Experiment 2 demonstrated that physical fatigue strongly altered these beneficial effects of dMI, as sMI_f_ further impacted performance, while dMI_f_ did not (as compared to the CONTROL_f_ condition). This result first supports the sMI efficacy on shooting tasks [54–57], and more specifically in free-throw shooting [32]. The superiority of sMI_f_ over dMI_f_ might be explained by the interference between actual and predicted body states in exhausted participants. Previous experiments showed that imagery ability could be degraded by physical fatigue [18, 19]. The authors inferred a possible erroneous update of the internal representation of the action due to fatigue. In our experiments, fatigue was elicited by a running test and athletes had to imagine a shooting task. However, as action representation was embodied and integrated the current state of the motor system [19], physical fatigue might have led to inappropriate state estimation provided by the forward model during dMI_f_. Physiological body state is known to affect body representation through subtle changes in proprioceptive inputs to the central nervous system [58]. In other words, physiological body state plays an indirect role during central processing of imagined actions, since these involve predictive models derived from the current state of the motor system [15]. Hence, combining body movements during dMI as athletes were fatigued possibly increased mismatches between actual and predictive body states. Practically, under fatigue, dMI may be assimilated to a form of incongruence between imagery task and body state [59].

One of the major finding of our experiments is that MI selectively contributed to enhance motor performance, with the optimal use of sMI under fatigue, concurrently with dMI being linked to exhaustion/energy expenditure. dMI might have higher abilities than sMI to improve movement accuracy when athletes are not fatigued, whereas under physical fatigue, sMI would be more efficient. Although the present study remains a pilot experiment with a limited sample size including only young athletes, it provides fruitful new insights about the optimal use of sMI and dMI. As preliminary recommendations, we argue that dMI may be prioritized in activities which do not lead to extreme effort and fatigue or in the early phases of the game, i.e. when athletes are not fatigued. In contrast, although dMI is not harmful or debilitative, this is not the most relevant alternative in a fatigued state or when athletes are exhausted. Conversely, sMI should thus be preferred in activities where accuracy is crucial and where fatigue can concurrently impair performance. To overcome the potential issue of the limited number of shooting trials in the present pilot experiment, future studies testing the effect of sMI/dMI practice before *each* free-throw may afford a greater number of trials, while concomitantly controlling ceiling effects due to the repeated practice of the skill. However, such experimental design would not match the demands of an actual basketball game where the number of consecutive shooting trials remains limited. An interesting perspective of the present work would be testing whether sMI and dMI might selectively impact recovery processes in a rehabilitation context according to different fatigue/functional levels.

## Supporting Information

S1 Dataset Raw data collected during the experiment. (CSV)
