# Correction: Selective Efficacy of Static and Dynamic Imagery in Different States of Physical Fatigue

**DOI:** 10.1371/journal.pone.0153622

**Published:** 2016-04-11

**Authors:** The PLOS ONE Staff

The first and third authors’ names appear incorrectly in the citation. The correct names are: Kanthack TFD and Altimari LR. The correct citation is: Kanthack TFD, Guillot A, Altimari LR, Nunez Nagy S, Collet C, Di Rienzo F (2016) Selective Efficacy of Static and Dynamic Imagery in Different States of Physical Fatigue. PLoS ONE 11(3): e0149654. doi:10.1371/journal.pone.0149654. The publisher apologizes for the errors.
